# Trends in the hospital-sector consumption of the WHO AWaRe Reserve group antibiotics in EU/EEA countries and the United Kingdom, 2010 to 2018

**DOI:** 10.2807/1560-7917.ES.2022.27.41.2101058

**Published:** 2022-10-13

**Authors:** Ria Benkő, Mária Matuz, Zoltán Pető, Klaus Weist, Ole Heuer, Vera Vlahović-Palčevski, Dominique L Monnet, Githa Fungie Galistiani, Hege Salvesen Blix, Gyöngyvér Soós, Edit Hajdú, Members of the ESAC-Net AWaRe study group, ESAC-Net AWaRe study group, Klaus Weist, Ole Heuer, Dominique L. Monnet, Ria Benkő, Mária Matuz, Githa Fungie Galistiani, Gyöngyvér Soós, Zoltán Pető, Edit Hajdu, Hege Salvesen Blix, Vera Vlahović-Palčevskih, Eline Vandael, Stefana Sabtcheva, Marina Payerl-Pal, Ute Wolff Sönksen, Elviira Linask, Emmi Sarvikivi, Philippe Cavalié, Birgitta Schweickert, Flora Kontopidou, Ágnes Hajdú, Ajay Oza, Filomena Fortinguerra, Andis Seilis, Jolanta Kuklytė, Marcel Bruch, Peter Zarb, Stephanie Natsch, Anna Olczak-Pieńkowska, Ana Silva, Tomáš Tesař, Milan Cižman, Jenny Hellman, Susan Hopkins

**Affiliations:** 1 University of Szeged, Faculty of Pharmacy, Institute of Clinical Pharmacy, Szeged, Hungary; 2 University of Szeged, Albert-Szent Györgyi Medical Centre, Central Pharmacy, Szeged, Hungary; 3 University of Szeged, Albert-Szent Györgyi Medical Centre, Emergency Department, Szeged, Hungary; 4 European Centre for Disease Prevention and Control (ECDC), Stockholm, Sweden; 5 Department of Clinical Pharmacology, University Hospital Rijeka/Medical Faculty and Faculty of Health Studies, University of Rijeka, Rijeka, Croatia; 6 WHO Collaborating Centre for Drug Statistics Methodology, Oslo, Norway; 7 Norwegian Institute of Public Health, Oslo, Norway; 8 The research group for personalized pharmacotherapy and clinical pharmacy, Department of Pharmacy, University of Oslo, Norway; 9 University of Szeged, Albert-Szent Györgyi Medical Centre, Internal Medicine Department, Infectious Disease Unit, Hungary; 10 Members of the ESAC-Net AWaRe study group are listed under Collaborators

**Keywords:** antibacterial use, hospital care sector, reserve group, antibiotic stewardship, public health, drug utilisation, DDD per 1,000 inhabitants per day, Europe

## Abstract

**Background:**

In 2019, the World Health Organization published the 21^st^ Model list of Essential Medicines and updated the Access, Watch Reserve (AWaRe) antibiotics classification to improve metrics and indicators for antibiotic stewardship activities. Reserve antibiotics are regarded as last-resort treatment options.

**Aim:**

We investigated hospital-sector consumption quantities and trends of Reserve group antibiotics in European Union/European Economic Area countries and the United Kingdom (EU/EEA/UK).

**Methods:**

Hospital-sector antimicrobial consumption data for 2010–2018 were obtained from the European Centre for Disease Prevention and Control. Antibacterials’ consumption for systemic use (Anatomical Therapeutic Chemical classification (ATC) group J01) were included in the analysis and expressed as defined daily doses (DDD) per 1,000 inhabitants per day. We defined reserve antibiotics as per AWaRe classification and applied linear regression to analyse trends in consumption of reserve antibiotics throughout the study period.

**Results:**

EU/EEA/UK average hospital-sector reserve-antibiotic consumption increased from 0.017 to 0.050 DDD per 1,000 inhabitants per day over the study period (p = 0.002). This significant increase concerned 15 countries. In 2018, four antibiotics (tigecycline, colistin, linezolid and daptomycin) constituted 91% of the consumption. Both absolute and relative (% of total hospital sector) consumption of reserve antibiotics varied considerably (up to 42-fold) between countries (from 0.004 to 0.155 DDD per 1,000 inhabitants per day and from 0.2% to 9.3%, respectively).

**Conclusion:**

An increasing trend in reserve antibiotic consumption was found in Europe. The substantial variation between countries may reflect the burden of infection with multidrug-resistant bacteria. Our results could guide national actions or optimisation of reserve antibiotic use.

## Introduction

Antimicrobial resistance (AMR) is a major global public health concern. The main driver of AMR development is the consumption of antimicrobial agents [1]. Overall, in the European Union/European Economic Area and the United Kingdom (EU/EEA/UK), it is estimated that nearly one third of patients admitted to acute care hospitals are receiving at least one antimicrobial agent [2]. The European Centre for Disease Prevention and Control (ECDC) estimated that, each year, there are ca 4.5 million healthcare-associated infections (HAIs) in acute care hospitals in the EU/EEA/UK. Approximately one-third of HAIs for which a microbiological result was obtained, was associated with antimicrobial-resistant bacteria [2,3].

Based on data from the European Antimicrobial Resistance Surveillance Network (EARS-Net) [4] and the 2011–2012 ECDC point prevalence survey (PPS) [5], ECDC estimated that more than 33,000 patients die each year in the EU/EEA/UK due to an infection with antimicrobial-resistant bacteria [6].

Due to a lack of new alternative antimicrobial agents, prudent use of existing antimicrobial agents remains crucial in the effort to prevent and control AMR [7,8]. In 2017, parallel to ‘the Model list of Essential Medicines’ (EML), the World Health Organization (WHO) published a classification of antibiotics, i.e. Access, Watch and Reserve antibiotics, which is referred to as the AWaRe [9,10]. This classification, which was updated in 2019, was developed to assist antibiotic stewardship programmes with global metrics and indicators for appropriate use [11]. To date there seems to be only one drug utilisation publication based on the 2019 AWaRe classification, focusing on combined community and hospital sector data, worldwide [12]. A few studies using the previous 2017 AWaRe classification framework have been reported, but these were more limited in geographical or patient population scope [13-18].

Within AWaRe, antibiotics are classified into three groups: the Access group, the Watch group and the Reserve group. The Access group comprises the recommended first or second choice for treatment of bacterial infections, offering the best therapeutic value, while minimising the potential for AMR [11]. Antibiotics in the Watch group are recommended as a first or second choice for empiric treatment of specific, limited number of infective syndromes, but have in general a strong potential for generating AMR in bacteria [11]. Finally, the Reserve group (Table) includes antibiotics and antibiotic classes that should be reserved for the treatment of confirmed or suspected infections due to multidrug-resistant (MDR) organisms (‘last-resort’ treatment option) [11]. Monitoring the use of reserve antibiotics is a key target of national and international stewardship programmes, in order to help preserve their effectiveness [11].

**Table:** Reserve group antibiotics WHO AWaRe class^a^, ATC classification code^b^, substance name, and occurrence of hospital consumption based on country-level reports, EU/EEA countries and the UK, 2010–2018 (n = 23 countries reporting)

Class^a^	ATC code^b^	Substance ATC name	Hospital consumption reported during 2010–2018
Tetracyclines	J01AA08	Minocycline^c^	Yes
	J01AA13	Eravacycline	No
	J01AA15	Omadacycline	No
Glycylcyclines	J01AA12	Tigecycline	Yes
Third-generation cephalosporins	J01DD52	Ceftazidime-avibactam	Yes
Fifth-generation cephalosporins	J01DI01	Ceftobiprole medocaril	Yes
	J01DI02	Ceftaroline fosamil	Yes^d^
	J01DI54	Ceftolozane-tazobactam	Yes
Penems	J01DI03	Faropenem	No
Carbapenems	J01DH52	Meropenem-vaborbactam	No
Monobactams	J01DF01	Aztreonam	Yes
Streptogramins	J01FG02	Dalfopristin-quinupristin	Yes^d^
Aminoglycosides	J01GB14	Plazomicin	No
Glycopeptides	J01XA03	Telavancin	No
	J01XA04	Dalbavancin	Yes
	J01XA05	Oritavancin	No
Polymyxins	J01XB01	Colistin	Yes
	J01XB02	Polymyxin B	Yes
Phosphonics	J01XX01	Fosfomycin^c^	Yes
Lipopeptides	J01XX09	Daptomycin	Yes
Oxazolidinones	J01XX08	Linezolid	Yes
	J01XX11	Tedizolid	Yes

ATC: Anatomical Therapeutic Chemical classification; AWaRe: Access, Watch and Reserve antibiotics; EEA: European Economic Area; EU: European Union; UK: United Kingdom; WHO: World Health Organization.

^a^ As defined in the WHO AWaRe classification in 2019 [11].

^b^ As defined in [21].

^c^ Minocycline and fosfomycin are only considered part of the Reserve group of antibiotics, if they are administered intravenously.

^d^ Marginal use.

Increasing use of reserve antibiotics may be regarded as an unavoidable consequence of increasing prevalence of antimicrobial-resistant bacteria, especially MDR bacteria, in hospitals or can indicate inadequate prescribing, or a mixture of both. The present study aims to describe the magnitude and trends in the consumption of reserve antibiotics overall and by class across the hospital sector in EU/EEA countries and the UK during the period 2010–2018.

## Methods

The study included countries that submitted hospital sector antimicrobial consumption data to the European Surveillance of Antimicrobial Consumption Network (ESAC-Net) and made their data available for analysis. Data referring to the study period 2010‒2018, and including information on national data sources (sales or reimbursement data or both), were retrieved from The European Surveillance System (TESSy) at ECDC [19]. In TESSy, only one dataset on national antibiotic consumption is uploaded per country [19]. The national datasets represent the best available national data sources, generally stable over time and providing representative hospital sector data with, ordinarily more than 95% coverage [20]. Entries within each dataset may originate from antibiotic sales, reimbursements of antibiotic purchases, or both, with possibility of alternating between years. For a few countries, which simultaneously collect sales and reimbursement data, the TESSy dataset can be based on both data types, whereby reimbursement data are used to validate sales data or vice‒versa [19].

Consumption data at substance level of antibacterials for systemic use according to the Anatomical Therapeutic Chemical classification (ATC code J01, 5^th^ group level), were expressed as defined daily doses (DDD) per 1,000 inhabitants per day, thus enabling comparison of national antimicrobial consumption data. The 2020 ATC/DDD index was applied to all data [21]. Only countries with available data on reserve antibiotics for at least four consecutive years were included in the trend analyses.

Based on the data obtained from the included countries, an estimate of the EU/EEA/UK average consumption, expressed as DDD per 1,000 inhabitants per day, was calculated as population-weighted mean. Linear regression was applied to analyse trends in consumption of reserve antibiotics throughout the study period. Trends were described by the regression coefficient (average annual change) and significance (p value) from the regression formula [19]. p value of < 0.05 was considered as significant.

For geographical comparison of the consumption of reserve antibiotics, countries where grouped into European sub-regions according to EuroVoc [22]. Statistics were calculated using MS Excel and R (version: 3.6) while graphics were created with MS Excel, the EMMA Map maker and R (Version 3.6 ggplot2 package) [23,24]. We correlated the consumption of reserve antibiotics in the hospital sector with the total systemic antibacterial use in the hospital sector using non-parametric Spearman’s rank correlation test.

## Results

**Figure 1:**
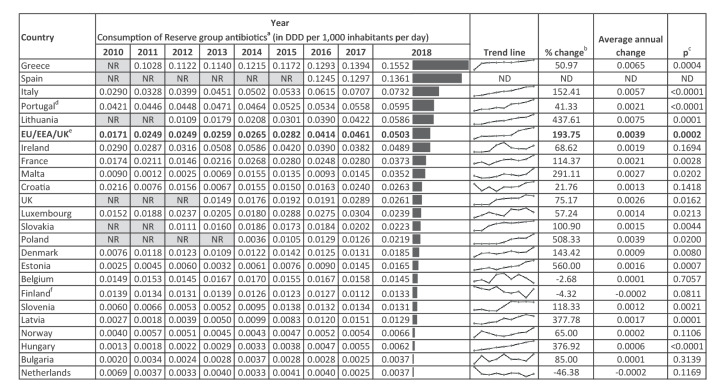
Hospital-sector consumption (in DDD per 1,000 inhabitants per day) of Reserve group antibiotics^a^, as well as changes in consumption over the study period, EU/EEA countries and the UK, 2010–2018 (n = 23 countries)

AWaRe: Access, Watch and Reserve antibiotics; DDD: defined daily doses; EEA: European Economic Area; EU: European Union; ND: not determined for countries reporting data for less than four consecutive years; NR: hospital care antibiotic consumption data not reported to The European Surveilllance System (TESSy); UK: United Kingdom; WHO: World Health Organization.

^a^ As defined in the WHO AWaRe classification [11].

^b^ Percentage of change between the last year of the study period and the earliest study year when data are available.

^c^ p < 0.05 was considered as significant.

^d^ Portugal: data refer to public hospitals.

^e^ Population-weighted mean based on data from countries listed in the Table.

^f^ Finland: data include consumption in remote primary healthcare centres and nursing homes.

A total of 23 countries (Belgium, Bulgaria, Croatia, Denmark, Estonia, Finland, France, Greece, Hungary, Ireland, Italy, Latvia, Lithuania, Luxembourg, Malta, the Netherlands, Norway, Poland, Portugal, Slovakia, Slovenia, Spain and the UK) provided hospital sector antimicrobial consumption data to TESSy and made them available for analysis. All of these countries reported consumption of reserve antibiotics (Table) and were included in the analysis. For the trend analysis, 22 countries that reported data for at least four consecutive years were included (Figure 1).

Fifteen countries (Bulgaria, Denmark, Estonia, Finland, France, Greece, Hungary, Latvia, Lithuania, Malta, the Netherlands, Norway, Poland, Slovakia and Spain) reported sales data during the entire study period. Among the remaining countries, Belgium, Croatia, and Italy reported reimbursement data, while Ireland and Portugal provided data based on sales and reimbursement data sources for the whole study period. Luxembourg changed data source, from reimbursement to sales data, in 2015. Slovenia reported data from both sales and reimbursement data sources from 2010 to 2012, and afterwards only reimbursement data, while the UK reported data from both sources, except for 2016 and 2017 when this country only reported reimbursement data.

**Figure 2:**
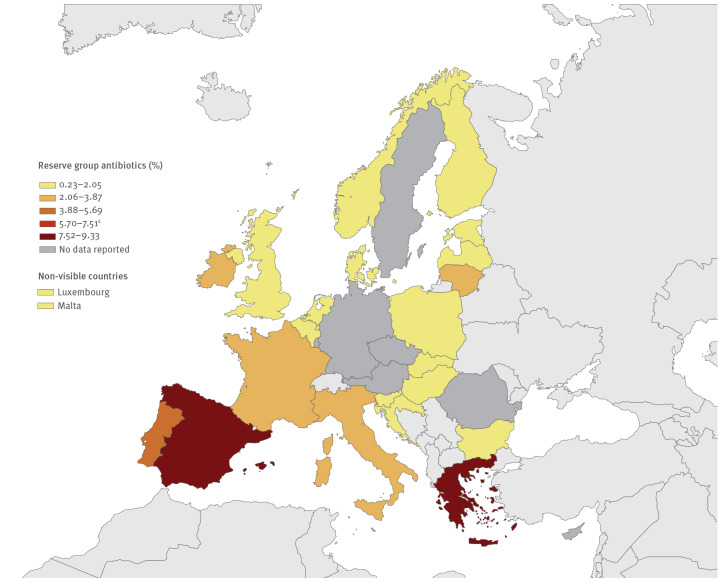
Countries according to proportions of Reserve group^a^ antibiotics among antibacterials consumed for systemic use^b^ in the hospital sector, EU/EEA countries and the UK, 2018 (n = 23 countries)

ATC: Anatomical Therapeutic Chemical classification; AWaRe: Access, Watch and Reserve antibiotics; EEA: European Economic Area; EU: European Union; UK: United Kingdom; WHO: World Health Organization.

^a^ As defined in the WHO AWaRe classification [11].

^b^ ATC J01.

^c^ No country had relative consumption of Reserve group antibiotics comprised between 5.70 and 7.51%.

**Figure 3:**
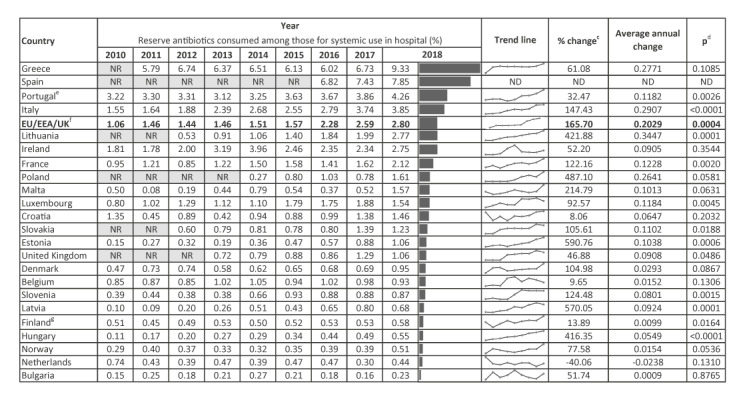
Yearly proportions of Reserve group^a^ antibiotics among antibacterials consumed for systemic use^b^ in the hospital sector, as well as changes over the study period, EU/EEA and the UK, 2010–2018 (n = 23 countries)

ATC: Anatomical Therapeutic Chemical classification; AWaRe: Access, Watch and Reserve antibiotics; EEA: European Economic Area; EU: European Union; ND: not determined for countries reporting data for less than four consecutive years; NR: hospital care antibiotic consumption data not reported to The European Surveilllance System (TESSy); UK: United Kingdom; WHO: World Health Organization.

^a^ As defined in the WHO AWaRe classification [11].

^b^ ATC J01.

^c^ Percentage of change between the last year of the study period and the earliest study year when data are available.

^d^ p < 0.05 was considered as significant.

^e^ Portugal: data refer to public hospitals.

^f^ UK and EU/EEA/UK countries’ population-weighted mean.

^g^ Finland: data include consumption in remote primary healthcare centres and nursing homes.

In 2018, a total of 7,025,162 DDDs of reserve antibiotics were consumed in the hospital sector in the 23 reporting countries. This corresponded to an estimated EU/EEA/UK average hospital-sector consumption of 0.050 DDD per 1,000 inhabitants per day, ranging from 0.004 in the Netherlands and Bulgaria to 0.155 in Greece, or a 42-fold difference (Figure 1, Figure 2). Of the five countries with higher consumption of reserve antibiotics than the EU/EEA/UK average, four were from southern Europe (Figure 1). The EU/EEA/UK average proportion of consumption of reserve antibiotics relative to the total hospital-sector consumption of antibacterials for systemic use was 2.8%, ranging from 0.2% in Bulgaria to 9.3% in Greece (Figure 2, Figure 3).

For Finland, data include consumption in remote primary healthcare centres and nursing homes. For Portugal, data refer to public hospitals.

Administrative boundaries: EuroGeographics, UN-FAO; Turkstat. The boundaries and names shown on this map do not imply official endorsement or acceptance by the European Union.

The EU/EEA/UK average consumption of reserve antibiotics increased significantly from 0.017 to 0.050 DDD per 1,000 inhabitants per day over the study period (p = 0.002) (Figure 1). A significant increase in the consumption of reserve antibiotics was observed in 15 countries (Denmark, Estonia, France, Greece, Hungary, Italy, Latvia, Lithuania, Luxembourg, Malta, Poland, Portugal, Slovakia, Slovenia and the UK), whereas none of the countries showed a significant decrease (Figure 1).

**Figure 4:**
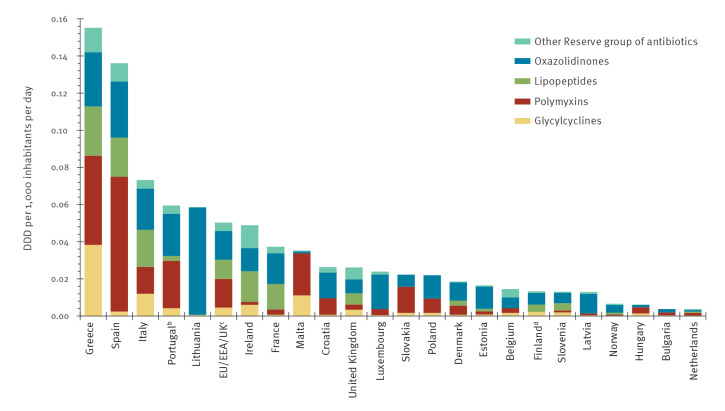
Hospital-sector consumption (in DDD per 1,000 inhabitants per day) of Reserve group antibiotics^a^, EU/EEA countries and the UK, 2018 (n = 23 countries)

AWaRe: Access, Watch and Reserve antibiotics; DDD: defined daily doses; EEA: European Economic Area; EU: European Union; UK: United Kingdom; WHO: World Health Organization.

^a^ As defined in the WHO AWaRe classification [11].

^b^ For Portugal, data refer to public hospitals.

^c^ UK and EU/EEA countries’ population-weighted mean.

^d^ For Finland, data include consumption in remote primary healthcare centres and nursing homes.

Of the 14 antibiotic classes in the Reserve group (Table), four classes (glycylcyclines, polymyxins, lipopeptides, and oxazolidinones) constituted the majority of the consumption (Figure 4). Together, these four antibiotic classes represented 91.0% of the average consumption of reserve antibiotics in the EU/EEA/UK in 2018 and made up more than 80% of the consumption of reserve antibiotics in most countries with the exception of Belgium, Ireland and the UK. The individual countries’ consumption of glycylcyclines, polymyxins, oxazolidinones and lipopeptides are provided in the Supplementary tables.

The EU/EEA/UK average consumption of glycylcyclines (the only substance in this class was tigecycline; Table S1) increased significantly from 0.003 to 0.005 DDD per 1,000 inhabitants and per day (p = 0.028) between 2010 and 2018. In 2018, the consumption of glycylcyclines was above the EU/EEA/UK average in Greece, Ireland, Italy and Malta. Significantly increasing trends (p < 0.05) in the consumption of glycylcyclines were observed in eight countries (Estonia, France, Greece, Hungary, Ireland, Italy, Malta, and the UK) during 2010–2018.

The EU/EEA/UK average consumption of polymyxins (almost exclusively colistin; Table S2) quadrupled between 2010 and 2018 (from 0.004 to 0.015 DDD per 1,000 inhabitants per day, p = 0.001). In 2018, consumption of polymyxins was the highest in Greece and Spain. At country-level, a significant increasing trend (p < 0.05) for consumption of polymyxins was observed in seven countries (Denmark, Greece, Hungary, Italy, Portugal, Slovakia and the UK) during 2010–2018 and consumption in all of these countries increased by more than 50% during this period.

The EU/EEA/UK average consumption of lipopeptides (the only substance in this class was daptomycin; Table S3) increased gradually and significantly between 2010 and 2018: from 0.002 to 0.01 DDD per 1,000 inhabitants per day (p < 0.0001). In 2018, lipopeptide consumption was the highest in Greece, Italy and Spain. A significant increasing trend (p < 0.05) was observed in seven countries (Denmark, France, Ireland, Italy, the Netherlands, Slovenia and the UK) during 2010–2018. Ten countries reported very low (< 0.001 DDD per 1,000 inhabitants per day) or no consumption of this class (Belgium, Bulgaria, Croatia, Hungary, Latvia, Lithuania, Luxembourg, Malta, Slovakia and Poland).

The EU/EEA/UK average consumption of oxazolidinones (nearly exclusively linezolid; Table S4) increased twofold (from 0.007 to 0.016 DDD per 1,000 inhabitants per day) between 2010 and 2018 (p = 0.0009), and Lithuania reported the highest consumption in 2018. In seven countries (Croatia, Estonia, Italy, Latvia, Lithuania, Luxembourg, Slovenia), oxazolidinone consumption increased significantly by more than 50% during 2010–2018.

The consumption of the various antibiotic classes within the Reserve group varied between countries. In 2018, the proportion of glycylcyclines (tigecycline) ranged from 31.5% (Malta) to 1.0% (Lithuania) of total consumption of antibacterials for systemic use (ATC J01). For polymyxins, the proportion was the highest in Malta (64.2%) and the lowest in Ireland (3.3%) (Figure 4). For oxazolidinones, this proportion ranged from 98.6% in Lithuania to 3.1% in Malta, while in countries with reported consumption of lipopeptides (daptomycin), the proportion ranged from 36.7% in France to 4.4% in Portugal.

No significant association was found, for EU/EEA/UK countries and in 2018, between the hospital-sector consumption of reserve antibiotics and the total hospital-sector consumption of antibacterials for systemic use (ATC J01) expressed in DDD per 1,000 inhabitants per day (data not shown).

## Discussion

This study provides an overview of the magnitude and trends in the hospital-sector consumption of reserve antibiotics in EU/EEA countries and the UK, based on the AWaRe classification [9]. Its strength is the nearly full population coverage of data in almost all of the studied countries, as well as the use of data that were continuously validated by national experts. National representatives from all reporting countries contributed to the interpretation of findings.

The estimated EU/EEA/UK average consumption of reserve antibiotics in the hospital sector was 0.05 DDD per 1,000 inhabitants per day in 2018, corresponding to approximatively 3% of consumption of antibacterials for systemic use (ATC J01) in this sector. This may appear as having low public health significance. However, assuming that the average duration of treatment with a reserve antibiotic could be between 8 to 12 days (1 course = 8 to 12 DDDs), the overall consumption of reserve antibiotics in the hospital sector would correspond to 790,000 to 1.18 million treatment courses administered in hospitals each year when extrapolated to the whole EU/EEA/UK, which poses a potential risk for the development of AMR in these patients and hospitals.

Based on the countries included in the study, the consumption of reserve antibiotics increased significantly in the EU/EEA/UK over the period 2010–2018. In 2018, there was also a large variation (ca 42-fold) in the consumption of reserve antibiotics between countries. Such a variation was not anticipated, as much smaller differences in the consumption of antibiotics for systemic use had been shown previously [25]. This may reflect the burden of AMR or the overuse/underutilisation of reserve antibiotics in individual countries. Another possible explanation is that low hospital consumption of reserve antibiotics expressed in DDD per 1,000 inhabitants per day, in e.g. Bulgaria, Hungary, the Netherlands and Norway (Figure 1), renders the annual maximum/minimum consumption sensitive to any increase or decrease in the use of Reserve group antibiotics (e.g. clusters of infections, drug availability, hospital/ambulatory care mix) and should be interpreted with caution.

We anticipated an association between the use of reserve antibiotics and the overall consumption of antibiotics for systemic use in hospitals, since high overall consumption – the most relevant risk factor for AMR ‒ would lead to high AMR rates and as a consequence lead to increased consumption of reserve antibiotics. However, we found no association between Reserve group consumption in hospitals and total hospital-sector consumption of antibacterials for systemic use (ATC J01). For example, the highest consumption of reserve antibiotics in 2018 was reported by Greece, although Greece reported a total hospital-sector consumption below the EU/EEA/UK average (data not shown). Inversely, the UK reported a low consumption of Reserve group antibiotics, but a high total hospital-sector consumption (data not shown). Patient-level data might have allowed to assess the appropriateness and quality of reserve antibiotic prescriptions, and in particular if they had been used as recommended as last-resort treatment options. The geographical variation found in this study, however, i.e. high consumption of reserve antibiotics in the hospital sector in southern European countries, is similar to that observed for total consumption of antibacterials for systemic use in the community (primary care) [26]. Such a geographical gradient has not been previously reported for the total consumption of antibacterials for systemic use in the hospital sector.

At substance level, the most frequently consumed reserve antibiotics were tigecycline, colistin, daptomycin and linezolid.

The EU/EEA/UK average consumption of tigecycline significantly increased during the study period. Greece had the highest consumption followed by Italy, Malta and Ireland. Within the reserve antibiotics, however, except for Greece, tigecycline consumption remained marginal in all countries. This can be explained by the narrow indications of tigecycline in Europe [27].

In 2018, the highest consumption of colistin in the EU/EEA/UK was reported by Spain followed by Greece. Colistin is used in hospitals to treat severe infections caused by MDR Gram-negative pathogens such as e.g. MDR *Pseudomonas aeruginosa*, *Acinetobacter* spp., *Klebsiella pneumoniae* that are resistant to carbapenems [28,29]. Data from EARS-Net show that the prevalence of hospital isolates of such pathogens was high in Spain (except *K. pneumoniae*) and in Greece, but also in other countries (e.g. Italy) [30,31], that reported a much lower consumption of colistin. This suggests that colistin might be used in Greece and Spain in more indications or higher doses than in other countries or that the burden of infections with MDR Gram-negative pathogens in Greece and Spain is higher than reported from just invasive isolates [25].

Regarding the trends, we observed a considerable (fourfold) increase in the use of colistin at the EU/EEA/UK level during the study period. A direct association between colistin use, emergence of colistin resistance and untreatable bacterial infections, has been reported [32]. However, the increased colistin consumption in the EU/EEA/UK can only be partly explained by AMR trends [25] as recent data from EARS-Net show an increasing trend at EU/EEA/UK level for combined AMR in *K. pneumoniae*, but not in other Gram-negative pathogens.

The primary indications of daptomycin in Europe are complicated skin and soft-tissue infections (cSSTI), bacteraemia or endocarditis [27] caused by MDR Gram-positive pathogens including meticillin-resistant staphylococci and vancomycin-intermediate or vancomycin-resistant *Staphylococcus aureus* (VISA and VRSA) [28]. It is also used off-label to treat vancomycin-resistant *Enterococcus* spp. (VRE) infections [28]. Daptomycin is also considered an alternative to linezolid. However, four of five countries with a consumption of daptomycin higher than the EU/EEA/UK average (France, Greece, Italy and Spain) also reported a consumption of linezolid higher than this average. In a context of decreasing meticillin-resistant *S.* *aureus* (MRSA) percentages and rare VISA and VRSA infections in the EU/EEA/UK, the observed significant increase of daptomycin consumption could reflect the increasing VRE percentages in several European countries [25].

Oxazolidinones, especially linezolid, serve as an alternative to vancomycin or daptomycin for the treatment of primarily infections caused by MRSA, other meticillin-resistant *Staphylococcus* spp. and vancomycin-resistant pathogens such as VRE. In 2018, Lithuania reported the highest absolute and relative consumptions of linezolid. This could possibly be explained by AMR trends, such as high percentages of VRE reported in Lithuania in the preceding years [25,30]. Another explanation could be that linezolid, instead of glycopeptides or daptomycin, is used for the treatment of MRSA infections. Daptomycin consumption was not reported from Lithuania, but glycopeptide consumption was moderate and showed an increasing trend (data not shown). In the EU/EEA/UK overall, VRE percentages increased from 5.6% in 2010 to 17.3% in 2018 [25], oxazolidinone (linezolid) consumption doubled during the same period.

Our study has some limitations. The majority of countries in the EU/EEA/UK reported hospital-sector consumption data for all years included in this study and from the same data source throughout the study period. However, when comparing results from sales or reimbursement data sources, reimbursement data are not including non-reimbursed antibiotic prescriptions. In most countries these differences would not substantially impact our comparisons. Only Luxembourg, changed the data source from reimbursement to sales data during the study period and trends in this country should be interpreted with caution.

In some countries, consumption of reserve antibiotics in the hospital sector could have been underestimated (for example, Italy only reported reimbursement data). On the other hand data on the hospital sector from Portugal only refer to public hospitals, which might lead to slight over or underestimation of actual antibiotic consumption. The differences in the used data sources (and consequent different drug coverage) might partly explain cross national differences in the use of reserve antibiotics, but has no influence on the observed trends of use.

In the calculations, we applied the DDD allocations valid from January 2020 onwards [21]. Therefore, any comparison with previously published reports on hospital antibiotic consumption in the EU/EEA/UK should be done with caution. Despite the revision of DDD values in the WHO ATC/DDD index 2019, in order to bring these values in agreement with current clinical practices (e.g. adult maintenance dose of parenteral colistin was changed from 3 to 9 million international units (IU) per day), the general limitations of the DDD methodology should be acknowledged. For example, for tigecycline, the DDD is still below the dosing recommended in the European Committee on Antimicrobial Susceptibility Testing (EUCAST) guidance on tigecycline dosing [33], which means that consumption of these reserve antibiotics expressed in DDD per 1,000 inhabitants per day may overestimate actual consumption.

As reserve antibiotics are indicated as last-resort option for treatment of infections caused by, or presumably caused by, MDR bacteria, and are mostly used in hospitals, this study focused on the hospital sector. Nevertheless, the definition of the hospital sector may differ between countries depending on the structure of the healthcare system. Moreover, certain reserve antibiotics (e.g. linezolid and colistin) may be used in primary care outside of the hospital sector and this may partly explain some of the differences between countries, e.g. for colistin. This means that our study did not assess the overall consumption of reserve antibiotics in the EU/EEA/UK.

The contribution of donated medicines to the consumption may be regarded as insignificant in the EU/EEA/UK. Likewise, the availability of specific antimicrobial agents may vary between countries, however in the EU/EEA/UK the effect of this limitation on consumption is likely to be low. Finally, results were not corrected for multiple testing, thereby increasing the risk of type I error.

## Conclusion

Prudent use of reserve antibiotics as defined by the AWaRe classification is of utmost importance to maintain their effectiveness to treat infected patients. The significantly increasing trends in the consumption of reserve antibiotics in most of countries, raises concerns. The observed findings might reflect the real burden of AMR among pathogens or inappropriate use, which would both need to be addressed. In the absence of clinical individual patient data such as treatment indication or duration of treatment, the appropriateness of consumption of reserve antibiotics in countries cannot be assessed.

## Supplementary Data

323000 Supplementary Material
