# Supplementary material for: Trends in the hospital-sector consumption of the WHO AWaRe Reserve group antibiotics in EU/EEA countries and the United Kingdom, 2010 to 2018
Source: Euro Surveill. 2022 Oct 13;27(41):2101058. doi: 10.2807/1560-7917.ES.2022.27.41.2101058 (PMC9562808; doi:10.2807/1560-7917.ES.2022.27.41.2101058)
Supplement: Supplementary Material [file 2101058_SupplementaryMaterial.pdf]

Supplementary tables for the Reserve group antibiotics subgroups.

This supplementary material is hosted by Eurosurveillance as supporting information alongside the article [Trends in the hospital-sector consumption of the WHO AWaRe Reserve group antibiotics in EU/EEA countries and the United Kingdom, 2010 to 2018], on behalf of the authors, who remain responsible for the accuracy and appropriateness of the content. The same standards for ethics, copyright, attributions and permissions as for the article apply. Supplements are not edited by Eurosurveillance and the journal is not responsible for the maintenance of any links or email addresses provided therein."

Table S1: Hospital sector consumption of glycylcyclines (tigecycline), EU/EEA, 2010-2018 (in DDD per 1,000 inhabitants per day)

Country	2010	2011	2012	2013	2014	2015	2016	2017	2018	Trend line	% change	Average annual change	p*
Greece	NR	0.0254	0.0289	0.0269	0.0270	0.0275	0.0329	0.0397	0.0383		50.79	0.0019	0.0061
Italy	0.0072	0.0070	0.0083	0.0093	0.0092	0.0098	0.0100	0.0116	0.0120		66.67	0.0006	<0.0001
Malta	0.0000	<0.0001	0.0008	0.0030	0.0056	0.0044	0.0031	0.0067	0.0111		1287.50	0.0013	0.0043
Ireland	0.0021	0.0015	0.0012	0.0011	0.0056	0.0041	0.0030	0.0035	0.0060		185.71	0.0005	0.0355
**EU/EEA†**	**0.0026**	**0.0038**	**0.0041**	**0.0038**	**0.0036**	**0.0037**	**0.0037**	**0.0044**	**0.0045**		**72.50**	**0.0001**	**0.0283**
Portugal§	0.0051	0.0031	0.0017	0.0013	0.0012	0.0016	0.0025	0.0034	0.0042		-17.65	<0.0001	0.9459
United Kingdom	NR	NR	NR	0.0020	0.0023	0.0023	0.0021	0.0029	0.0034		70.00	0.0002	0.0318
Spain	NR	NR	NR	NR	NR	NR	0.0034	0.0028	0.0024				
Finland‡	0.0043	0.0031	0.0022	0.0024	0.0022	0.0019	0.0022	0.0018	0.0022		-48.84	-0.0002	0.0192
Slovenia	0.0017	0.0009	0.0008	0.0010	0.0010	0.0018	0.0020	0.0027	0.0019		11.76	0.0002	0.0502
Belgium	0.0032	0.0032	0.0026	0.0022	0.0022	0.0021	0.0022	0.0023	0.0017		-46.88	-0.0002	0.0023
Poland	NR	NR	NR	NR	0.0010	0.0011	0.0001	0.0014	0.0017		70.00	0.0002	0.4514
Slovakia	NR	NR	0.0006	0.0012	0.0019	0.0019	0.0034	0.0031	0.0017		183.33	0.0003	0.0992
Hungary	0.0005	0.0005	0.0006	0.0008	0.0008	0.0010	0.0013	0.0015	0.0014		180.00	0.0001	<0.0001
Estonia	0.0001	0.0002	0.0005	0.0003	0.0004	0.0005	0.0007	0.0006	0.0008		700.00	0.0001	0.0006
Croatia	0.0012	0.0003	0.0002	0.0000	0.0028	0.0009	0.0005	0.0006	0.0007		-41.67	<0.0001	0.9554
Denmark	0.0004	0.0002	0.0002	0.0003	0.0004	0.0006	0.0004	0.0003	0.0007		75.00	<0.0001	0.0919
France	0.0006	0.0005	0.0005	0.0005	0.0006	0.0006	0.0006	0.0007	0.0007		16.67	<0.0001	0.0177
Lithuania	NR	NR	0.0002	0.0003	0.0003	0.0004	0.0007	0.0003	0.0006		200.00	0.0001	0.0954
Luxembourg	0.0020	0.0006	0.0014	0.0017	0.0013	0.0019	0.0006	0.0009	0.0005		-75.00	-0.0001	0.1627
Bulgaria	0.0000	0.0008	0.0011	0.0014	0.0014	0.0003	0.0003	0.0003	0.0004		-50.00	<0.0001	0.5582
Latvia	0.0006	0.0002	0.0002	0.0001	0.0001	0.0001	0.0002	0.0003	0.0004		-33.33	<0.0001	0.7254
Netherlands	0.0002	0.0001	0.0001	0.0002	0.0001	0.0002	0.0003	0.0002	0.0003		50.00	<0.0001	0.0622
Norway	0.0003	0.0002	0.0002	0.0003	0.0003	0.0002	0.0002	0.0002	0.0002		-33.33	<0.0001	0.2168

*Statistical significance of trends. †EU/EEA population-weighted mean. ‡Finland: data include consumption in remote primary healthcare centres and nursing homes. §Portugal: data refer to public hospitals. NR: not reported hospital care antibiotic consumption data to The European Surveilllance System (TESSy))

Table S2: Hospital sector consumption of polymyxins (colistin and polymyxin B), EU/EEA, 2010-2018 (in DDD per 1,000 inhabitants per day)

Country	2010	2011	2012	2013	2014	2015	2016	2017	2018		Trend line	% change	Average annual change	p*
Spain	NR	NR	NR	NR	NR	NR	0.0632	0.0703	0.0726					
Greece	NR	0.0286	0.0312	0.0313	0.0357	0.0372	0.0409	0.0448	0.0480			67.83	0.0028	<0.0001
Portugal§	0.0129	0.0185	0.0189	0.0197	0.0188	0.0218	0.0221	0.0228	0.0255			97.67	0.0012	0.0004
Malta	0.0087	0.0012	0.0006	0.0020	0.0036	0.0064	0.0050	0.0060	0.0226			159.77	0.0014	0.1105
**EU/EEA†**	**0.0038**	**0.0053**	**0.0067**	**0.0060**	**0.0059**	**0.0071**	**0.0143**	**0.0153**	**0.0154**			**305.24**	**0.0015**	**0.0009**
Italy	0.0047	0.0066	0.0099	0.0119	0.0134	0.0150	0.0143	0.0186	0.0144			206.38	0.0014	0.0007
Slovakia	NR	NR	0.0066	0.0075	0.0082	0.0084	0.0090	0.0123	0.0140			112.12	0.0012	0.0022
Croatia	0.0184	0.0033	0.0097	0.0010	0.0064	0.0061	0.0057	0.0089	0.0089			-51.63	-0.0004	0.5632
Poland	NR	NR	NR	NR	0.0002	0.0067	0.0113	0.0077	0.0077			3750.00	0.0016	0.2590
Denmark	0.0021	0.0018	0.0020	0.0026	0.0040	0.0035	0.0038	0.0038	0.0049			133.33	0.0004	0.0005
Hungary	0.0008	0.0013	0.0016	0.0021	0.0025	0.0028	0.0032	0.0033	0.0033			312.50	0.0003	<0.0001
Luxembourg	0.0034	0.0018	0.0016	0.0018	0.0011	0.0044	0.0016	0.0018	0.0032			-5.88	<0.0001	0.8479
France	0.0034	0.0025	0.0036	0.0037	0.0036	0.0036	0.0032	0.0014	0.0028			-17.65	-0.0001	0.2868
United Kingdom	NR	NR	NR	0.0017	0.0022	0.0023	0.0022	0.0023	0.0028			64.71	0.0002	0.0247
Belgium	0.0026	0.0029	0.0028	0.0032	0.0031	0.0028	0.0035	0.0032	0.0025			-3.85	<0.0001	0.5811
Estonia	0.0001	0.0004	0.0021	0.0013	0.0024	0.0031	0.0015	0.0033	0.0017			1600.00	0.0003	0.0556
Ireland	0.0046	0.0048	0.0048	0.0050	0.0043	0.0028	0.0019	0.0016	0.0016			-65.22	-0.0005	0.0007
Bulgaria	0.0000	0.0000	0.0000	0.0000	0.0007	0.0012	0.0013	0.0008	0.0015			114.29	0.0001	0.3268
Netherlands	0.0053	0.0024	0.0020	0.0022	0.0021	0.0026	0.0023	0.0008	0.0014			-73.58	-0.0003	0.0305
Slovenia	0.0004	0.0009	0.0012	0.0008	0.0016	0.0018	0.0027	0.0021	0.0010			150.00	0.0002	0.0685
Latvia	0.0000	0.0000	0.0009	0.0007	0.0004	0.0001	0.0008	0.0019	0.0009			0.00	0.0001	0.3937
Norway	<0.0001	0.0003	0.0004	0.0003	0.0005	0.0005	0.0004	0.0003	0.0006					
Finland‡	0.0000	0.0000	0.0000	0.0000	0.0000	0.0000	0.0000	0.0001	0.0000					
Lithuania	NR	NR	0.0000	0.0000	0.0000	0.0000	0.0000	0.0000	<0.0001					

*Statistical significance of trends. †EU/EEA population-weighted mean. ‡Finland: data include consumption in remote primary healthcare centres and nursing homes. §Portugal: data refer to public hospitals. NR: not reported hospital care antibiotic consumption data to The European Surveilllance System (TESSy))

Table S3: Consumption of lipopeptides (daptomycin), EU/EEA, 2010-2018 (in DDD per 1,000 inhabitants per day)

Country	2010	2011	2012	2013	2014	2015	2016	2017	2018		Trend line	% change	Average annual change	p*
Greece	NR	0.0210	0.0226	0.0253	0.0282	0.0265	0.0265	0.0255	0.0265			26.19	0.0007	0.0623
Spain	NR	NR	NR	NR	NR	NR	0.0189	0.0179	0.0211					
Italy	0.0065	0.0076	0.0092	0.0115	0.0133	0.0133	0.0147	0.0170	0.0200			207.69	0.0016	<0.0001
Ireland	0.0052	0.0055	0.0067	0.0098	0.0131	0.0143	0.0134	0.0138	0.0165			217.31	0.0015	0.0001
France	0.0005	0.0009	0.0014	0.0017	0.0079	0.0091	0.0111	0.0132	0.0137			2640.00	0.0019	<0.0001
**EU/EEA†**	**0.0021**	**0.0038**	**0.0043**	**0.0049**	**0.0062**	**0.0064**	**0.0085**	**0.0091**	**0.0103**			**391.24**	**0.0010**	**<0.0001**
United Kingdom	NR	NR	NR	0.0038	0.0044	0.0047	0.0049	0.0050	0.0060			57.89	0.0004	0.0033
Slovenia	0.0016	0.0021	0.0005	0.0014	0.0031	0.0043	0.0035	0.0031	0.0040			150.00	0.0004	0.0169
Finland‡	0.0029	0.0030	0.0047	0.0058	0.0050	0.0044	0.0037	0.0032	0.0039			34.48	0.0000	0.8873
Denmark	0.0005	0.0003	0.0004	0.0005	0.0007	0.0008	0.0010	0.0016	0.0027			440.00	0.0002	0.0041
Portugal§	0.0014	0.0028	0.0025	0.0030	0.0034	0.0039	0.0030	0.0026	0.0026			85.71	0.0001	0.2795
Estonia	0.0000	0.0000	0.0000	0.0000	0.0000	0.0000	0.0001	0.0000	0.0014					
Norway	0.0001	0.0004	0.0008	0.0008	0.0004	0.0006	0.0006	0.0007	0.0010			900.00	0.0001	0.0525
Netherlands	0.0003	0.0003	0.0003	0.0006	0.0003	0.0003	0.0005	0.0006	0.0006			100.00	0.0000	0.0448
Croatia	0.0003	0.0003	0.0001	0.0000	0.0000	0.0000	0.0000	0.0000	0.0000					
Luxembourg	0.0000	0.0007	0.0004	<0.0001	0.0000	<0.0001	0.0000	0.0000	0.0000					
Slovakia	NR	NR	<0.0001	<0.0001	<0.0001	<0.0001	0.0000	0.0000	0.0000					
Bulgaria	0.0001	<0.0001	0.0000	0.0000	0.0000	0.0000	0.0000	0.0000	0.0000					
Poland	NR	NR	NR	NR	0.0000	0.0000	0.0000	<0.0001	<0.0001					
Malta	0.0000	0.0000	0.0000	0.0000	0.0000	0.0000	0.0000	0.0000	0.0000					
Belgium	0.0000	0.0000	0.0000	0.0000	0.0000	0.0000	0.0000	0.0000	0.0000					
Hungary	0.0000	0.0000	0.0000	0.0000	0.0000	0.0000	0.0000	0.0000	0.0000					
Latvia	0.0000	0.0000	0.0000	0.0000	0.0000	0.0000	0.0000	0.0000	0.0000					
Lithuania	NR	NR	0.0000	0.0000	0.0000	0.0000	0.0000	0.0000	0.0000					

*Statistical significance of trends. †EU/EEA population-weighted mean. ‡Finland: data include consumption in remote primary healthcare centres and nursing homes. §Portugal: data refer to public hospitals NR: not reported hospital care antibiotic consumption data to The European Surveilllance System (TESSy))

Table S4: Hospital sector consumption of oxazolidinones (linezolid and tedizolid), EU/EEA, 2010-2018 (in DDD per 1,000 inhabitants per day)

Country	2010	2011	2012	2013	2014	2015	2016	2017	2018		Trend line	% change	Average annual change	p*
Lithuania	NR	NR	0.0107	0.0176	0.0205	0.0297	0.0383	0.0419	0.0578			440.19	0.0074	0.0001
Spain	NR	NR	NR	NR	NR	NR	0.0312	0.0300	0.0302					
Greece	NR	0.0229	0.0253	0.0245	0.0266	0.0256	0.0271	0.0255	0.0292			27.51	0.0006	0.0145
Portugal§	0.0225	0.0200	0.0213	0.0217	0.0210	0.0231	0.0232	0.0236	0.0227			0.89	0.0003	0.0620
Italy	0.0104	0.0112	0.0122	0.0123	0.0142	0.0151	0.0160	0.0194	0.0222			113.46	0.0014	0.0001
Luxembourg	0.0081	0.0130	0.0152	0.0154	0.0140	0.0176	0.0198	0.0223	0.0186			129.63	0.0014	0.0014
France	0.0082	0.0092	0.0056	0.0106	0.0109	0.0116	0.0084	0.0104	0.0166			102.44	0.0007	0.0522
**EU/EEA†**	**0.0070**	**0.0090**	**0.0083**	**0.0090**	**0.0089**	**0.0092**	**0.0115**	**0.0125**	**0.0155**			**120.44**	**0.0008**	**0.0011**
Croatia	0.0017	0.0037	0.0056	0.0057	0.0063	0.0080	0.0095	0.0115	0.0138			711.76	0.0014	<0.0001
Ireland	0.0150	0.0130	0.0147	0.0278	0.0297	0.0145	0.0142	0.0125	0.0125			-16.67	-0.0004	0.6504
Poland	NR	NR	NR	NR	0.0024	0.0027	0.0015	0.0035	0.0125			420.83	0.0021	0.1571
Estonia	0.0023	0.0039	0.0033	0.0015	0.0032	0.0039	0.0066	0.0105	0.0119			260.61	0.0011	0.0051
Latvia	0.0021	0.0016	0.0028	0.0042	0.0093	0.0080	0.0104	0.0117	0.0106			278.57	0.0014	0.0002
Denmark	0.0046	0.0064	0.0066	0.0075	0.0071	0.0088	0.0071	0.0072	0.0098			48.48	0.0004	0.0106
United Kingdom	NR	NR	NR	0.0050	0.0055	0.0056	0.0057	0.0063	0.0075			50.00	0.0004	0.0098
Slovakia	NR	NR	0.0039	0.0072	0.0085	0.0070	0.0060	0.0047	0.0065			66.67	<0.0001	0.9748
Finland‡	0.0062	0.0068	0.0057	0.0054	0.0049	0.0057	0.0061	0.0054	0.0063			1.61	<0.0001	0.5794
Belgium	0.0073	0.0073	0.0066	0.0068	0.0070	0.0061	0.0063	0.0058	0.0058			-20.55	-0.0002	0.0006
Slovenia	0.0022	0.0027	0.0027	0.0019	0.0035	0.0054	0.0048	0.0052	0.0056			154.55	0.0005	0.0013
Norway	0.0034	0.0046	0.0033	0.0026	0.0026	0.0032	0.0037	0.0038	0.0041			20.59	<0.0001	0.7486
Bulgaria	0.0015	0.0022	0.0011	0.0014	0.0016	0.0013	0.0012	0.0014	0.0018			20.00	<0.0001	0.7007
Hungary	<0.0001	<0.0001	<0.0001	0.0000	<0.0001	<0.0001	0.0002	0.0006	0.0012					
Malta	0.0000	<0.0001	0.0011	0.0015	0.0009	0.0013	0.0007	0.0011	0.0011				0.0001	0.3752
Netherlands	0.0010	0.0009	0.0008	0.0009	0.0007	0.0009	0.0008	0.0008	0.0009				<0.0001	0.3380

*Statistical significance of trends. †EU/EEA population-weighted mean. ‡Finland: data include consumption in remote primary healthcare centres and nursing homes. §Portugal: data refer to public hospit*als. NRl:* not reported hospital care antibiotic consumption data to The European Surveilllance System (TESSy))
